# Erlotinib-Loaded Dendrimer Nanocomposites as a Targeted Lung Cancer Chemotherapy

**DOI:** 10.3390/molecules28093974

**Published:** 2023-05-08

**Authors:** Wafa K. Fatani, Fadilah S. Aleanizy, Fulwah Y. Alqahtani, Mohammed M. Alanazi, Abdullah A. Aldossari, Faiyaz Shakeel, Nazrul Haq, Hosam Abdelhady, Hamad M. Alkahtani, Ibrahim A. Alsarra

**Affiliations:** 1 Department of Pharmaceutics, College of Pharmacy, King Saud University, Riyadh 11451, Saudi Arabia; 2 Department of Pharmacology and Toxicology, College of Pharmacy, King Saud University, Riyadh 11451, Saudi Arabia; 3 Department of Physiology & Pharmacology, College of Osteopathic Medicine, Sam Houston State University, 925 City Central Avenue, Conroe, TX 77304, USA; 4 Department of Pharmaceutical Chemistry, College of Pharmacy, King Saud University, Riyadh 11451, Saudi Arabia

**Keywords:** polyamidoamine dendrimers, erlotinib, non-small-cell lung cancer, cytotoxicity

## Abstract

Lung cancer is the main cause of cancer-related mortality globally. Erlotinib is a tyrosine kinase inhibitor, affecting both cancerous cell proliferation and survival. The emergence of oncological nanotechnology has provided a novel drug delivery system for erlotinib. The aims of this current investigation were to formulate two different polyamidoamine (PAMAM) dendrimer generations—generation 4 (G4) and generation 5 (G5) PAMAM dendrimer—to study the impact of two different PAMAM dendrimer formulations on entrapment by drug loading and encapsulation efficiency tests; to assess various characterizations, including particle size distribution, polydispersity index, and zeta potential; and to evaluate in vitro drug release along with assessing in situ human lung adenocarcinoma cell culture. The results showed that the average particle size of G4 and G5 nanocomposites were 200 nm and 224.8 nm, with polydispersity index values of 0.05 and 0.300, zeta potential values of 11.54 and 4.26 mV of G4 and G5 PAMAM dendrimer, respectively. Comparative in situ study showed that cationic G4 erlotinib-loaded dendrimer was more selective and had higher antiproliferation activity against A549 lung cells compared to neutral G5 erlotinib-loaded dendrimers and erlotinib alone. These conclusions highlight the potential effect of cationic G4 dendrimer as a targeting-sustained-release carrier for erlotinib.

## 1. Introduction

Cancer is the second largest cause of death worldwide. Among different types of cancer, lung cancer is considered the most common source of cancer-related death [1]. Among all lung cancers, non-small cell lung cancer (NSCLC) is responsible for 90% of cases, where the majority are found to be in a late stage when diagnosed [2]. Epidermal growth factor receptor (EGFR) is strongly expressed in NSCLC. Therefore, extensive efforts have been focused on the involvement of tyrosine kinase inhibitors of EGFR in the treatment of NSCLC [3,4]. Erlotinib (ERL), the EGFR tyrosine kinase inhibitor (TKI), is a quinazoline derivative targeted antiplatelet drug. It was approved in 2004 as a second-line treatment of NSCLC irrespective of EGFR genotype and a first-line treatment of activated mutations in EGFR [5]. ERL exhibits its antitumor activity by selectively occupying the adenosine triphosphate (ATP)-binding sites of EGFR, thus reversibly hindering EGFR activation. This leads to it suppressing the downstream signaling pathways, mainly by the two pathways of phosphatidylinositol 3-kinase (PI3K) and mitogen activated protein kinase (MAPK), and thus inhibiting cancer cell proliferation, angiogenesis, and metastases [6,7]. 

The aqueous solubility of ERL hydrochloride depends on pH, with an increase in solubility at a pH that is lower than 5, where maximal solubility of approximately 0.4 mg/mL arises at a pH of 2 [8]. Therefore, any alteration in the pH of the upper gastrointestinal (GI) tract may affect ERL solubility [9]. Since ERL has low aqueous solubility and high permeability (logP of 2.7), ERL is classified as class II in the biopharmaceutical classification (BCS) system [8]. In fact, many of these limitations are associated with ERL especially as the marketed ERL is administered orally as a film-coated tablet (Tarceva^®^, Astellas Pharm Global Development, Inc., Tokyo, Japan). These limitations include side effects, pH-dependent solubility, low permeability, drug interaction, and drug resistance. ERL’s poor solubility constrains its dissolution in the GI fluid, which restricts its absorption, thus resulting in a rise in its peak plasma concentration (C_max_) and area under the drug concentration–time curve (AUC), causing large inter-patient variability [10]. Moreover, food has been found to raise the bioavailability of the oral dosage form upon the administration of a 150 mg tablet for a level up to 100% from 60% in healthy volunteers, taken without food [11]. Additionally, studies showed that ERL was metabolized by the human liver primarily by CYP3A4, with a percentage of 70%, along with a secondary contribution from CYP1A2 of approximately 20%. For that reason, various factors that affect these hepatic enzymes may have an impact on ERL plasma concentration level [12,13]. 

Genomic variability between some ethnicities, drugs which either induce or reduce the enzymes, and smoking cigarettes are all factors that affect CYP3A4 and CYP1A2 levels and thus ERL plasma concentration levels [9]. Although ERL is similar to other chemotherapies in effectiveness, receivers of this medication were still experiencing ERL resistance after one year of treatment [14]. In addition, severe toxicities may appear, such as rashes, diarrhea, GI perforations, and Stevens–Johnson syndrome, which hamper its clinical application [15]. The pharmacokinetic parameters of the marketed tablet, Tarceva^®^, include a T_max_ of four hours and an elimination half-life of 36.2 h. All these limitations necessitate the willingness to overcome these obstacles by using proper nano delivery systems to enhance ERL efficacy and decrease undesirable adverse events and resistance. The proposed formulation can be assessed for future administration as an inhaled drug delivery system. This allows targeted drug delivery with minimized systemic side effects. 

Dendrimers are nanosized composite materials and known as dendrimer nanocomposites. To this end, polyamidoamine (PAMAM) dendrimers have emerged as promising, well-defined nanocarriers for targeted anticancer drug delivery; they have exceptional mono-dispersibility, nano-size, biocompatibility, the ability to enhance drug solubility, along with permeability, and retention. These dendrimers originate from an ethylene di-amine core; different dendritic branches are then inserted by exhaustive Michael addition, depending on the chosen number of generation (G0–G10) [16]. Dendrimers possess a unique molecular architecture consisting of a central core, dendrons, and peripheral functional groups, located on the outer surface of the macromolecule, that control DNA formation, drug encapsulation efficacy, and cellular targeting. Cellular ligands such as folic acid, hyaluronic acid, transferrin, peptides, and antibodies have been extensively applied for the development of tumor-selective drug delivery systems [16,17]. The dendrimer architecture has three main mechanisms for drug loading, namely, molecular entrapment within (i) the dendritic nanocapsules; (ii) branching points (making hydrogen bonds between dendrons and drug molecules); (iii) and external surface groups by electrostatic interactions [18,19]. The binding strength (H-8 is 100% and H-2 is 70%) of the encapsulated 6-mercaptopurine (6-MP) within the nanocavities of the amine-terminated dendrimers was quantitatively expressed using epitope maps [20]. Furthermore, the binding constants (logKa 3.85–4.74) of internal nanocavities of dendrimers and catecholamines were measured by UV–Vis, fluorescence, and 1D and 2D NMR spectroscopy [21]. A study was conducted by including the anticancer drug tetra methyl scutellarein entrapped inside a PAMAM fourth generation (G4) dendrimer [22]. The results showed that the blank PAMAM G4 dendrimer enhanced the water solubility of the medication. In addition, enhancement in encapsulation efficiency with a percentage 77.8 ± 0.69% and drug loading rates with a percentage 6.2 ± 0.06% were observed [22]. Another study showed that a multifunctional drug delivery system was developed to co-deliver ERL in combination with gene-recombinant short hairpin RNA-expressing plasmids (Survivin-shRNA) in modified PAMAM with chloroquine [23]. Chloroquine was added to enhance the endosomal escape capability of the AP/ES gene for effective gene transfection to obstruct survivin, where downregulation of survivin may overcome epidermal growth factor receptor-tyrosine kinase inhibitor resistance (EGFR-TKI). Additionally, it exhibited high vessel-normalization, which increases tumor microcirculation, which then promotes drug delivery to cancerous cells and improves ERL efficacy, especially in ERL-resistant cancer cells [24]. Other supramolecular drug delivery systems have also been investigated for cancer targeting and other therapeutic applications [25,26,27,28]. The aim of this work is to design non-invasive erlotinib nanocomposites using polyamidoamine dendrimers for the treatment of non-small cell lung cancer.

## 2. Results

### 2.1. HPLC Analysis of ERL

The proposed HPLC method for ERL analysis was linear at the range of 1–100 μg/mL. The linear regression equation was predicted as y = 50,678x + 4472, in which x is the concentration of ERL and y represents the measured peak area for ERL. The determination coefficient (R^2^) for ERL was found to be 0.991. The accuracy of the proposed HPLC method for ERL analysis was estimated as 98.21–101.43%. The intra-day and intermediate precision values for the anticipated HPLC technique were estimated as 0.68–1.23 and 0.74–1.42%, respectively. The relative standard deviation (% RSD) for robustness analysis was found to be 0.62–0.97%. The limit of detection (LOD) and limit of quantification (LOQ) values were determined to be 0.38 μg/mL and 1.14 μg/mL, respectively. The previous findings suggested that the proposed HPLC method for the determination of ERL analysis was linear, accurate, precise, robust, and sensitive. 

### 2.2. Formulation, Loading and Characterization of ERL in PAMAM Dendrimers

**Figure 1:**
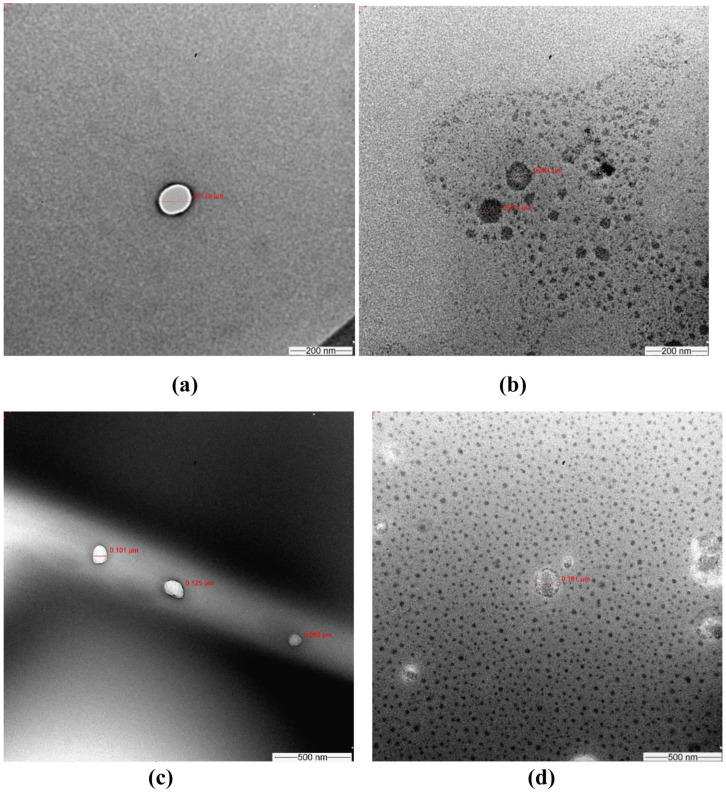
TEM images at higher magnifications to inspect the morphology of (**a**) blank Generation 4-FITC (G4-FITC) polyamidoamine (PAMAM) dendrimers; (**b**) erlotinib conjugated G4-FITC PAMAM dendrimers proving erlotinib placement within the dendrimer; (**c**) blank generation (G5-FITC) PAMAM dendrimers; and (**d**) erlotinib conjugated G5-FITC PAMAM dendrimers proving encapsulation of erlotinib within the dendrimer cavity.

**Figure 2:**
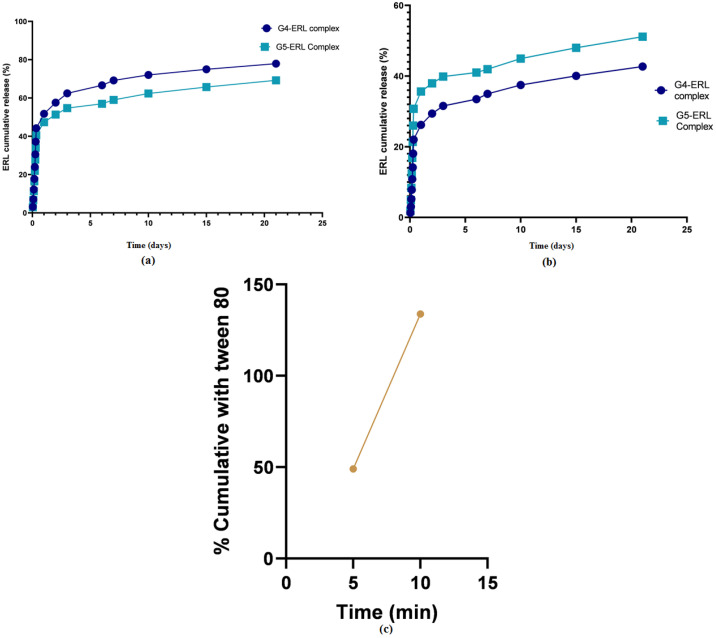
In vitro release of erlotinib, assessed as a percentage of cumulative release from dendrimers G4-FITC and G5-FITC, showing comparable prolonged release patterns. (**a**) Comparative in vitro release study at pH 5.4 for PAMAM dendrimer G4-FITC and G5-FITC; (**b**) comparative in vitro release study at pH 7.4 for PAMAM dendrimer G4-FITC and G5-FITC; and (**c**) comparison in vitro release study for ERL tablet between two media: 0.01 N HCl and 0.02% Tween 80 in 0.01 N HCl.

ERL was inserted individually into G4-FITC and G5-FITC PAMAM dendrimers, after which it was lyophilized. Data from previous reports have been considered to choose the most fit molar ratio of ERL to G4-FITC and G5-FITC PAMAM dendrimers. The chosen molar ratio of ERL to dendrimer was 25:1 [29]. Transmission Electron Microscope (TEM) imaging was utilized for morphological characterization of the blank and conjugated dendrimers. TEM images for different dendrimers at higher magnifications are presented in Figure 1. The TEM images for different dendrimers were also recorded at low magnifications and results are shown in Figure S1. The calculated percentage amount of encapsulation efficiency (EE) was 99.96% and 99.92% for formulations G4-FITC and G5-FITC, respectively. However, the percentage amount of drug-loading capacity (DLC) was 14.05% and 7.52% for formulations G4-FITC and G5-FITC, respectively. Figure 2 illustrates the in vitro release of ERL from G4-FITC and G5-FITC ERL-loaded dendrimers in addition to 150 mg ERL tablets. The two generations of PAMAM dendrimer formulations revealed sustained release patterns up to seven days while the tablet was released after 10 min. 

The physicochemical characteristics of blank and ERL-loaded PAMAM dendrimer G4-FITC and G5-FITC, such as particle size and polydispersity index (PDI), were measured (Table 1). The particle size distribution by intensity for blank and ERL-loaded G4-FITC and G5-FITC dendrimers are presented in Figure S2. Additionally, the exterior charge found on the surface of the dendrimers was estimated at various stages and environmental conditions. The zeta potential values for blank and ERL-loaded G4-FITC and G5-FITC dendrimers at pH 5.4 and 7.4 are presented in Figure S3 and Figure S4, respectively. The cationic G4-FITC plain dendrimer demonstrated a positive zeta potential in phosphate buffer solution (PBS) pH 5.4 and pH 7.4 (8.9 ± 1.1 and 10.6 ± 0.1 mV), respectively. These values were expected to be from the cationic surface groups on their surface due to the positive net charge. After loading with ERL, the zeta potential increased to 15.7 ± 1.34 and 18.2 ± 1.9 mV. Meanwhile, the neutral G5-FITC plain dendrimer demonstrated a negative zeta potential in PBS pH 5.4 and pH 7.4 (−5.66± 0.76 and −10.2 ± 0.21 mV). These values were expected to be from the anionic surface groups on their surface due to the negative net charge. After loading with ERL, the zeta potential value increased to −3.82 ± 0.66 and −5.78 ± 0.7 mV, respectively. In previous studies, the zeta potential of plain G5 PAMAM dendrimers was also recorded as a negative value [29]. After loading a weak base drug, such as ruboxistaurin, the zeta potential value of G5 dendrimers was increased [29]. Another study also indicated the increased zeta potential of G5 dendrimers after loading a weak base drug, namely, vardenafil [30]. In the present study, the selected drug ERL is a weak base. The zeta potential of G5-FITC dendrimers was also increased after drug loading in the present study. Therefore, the obtained results of plain and drug-loaded FITC-G5-dendrimers were in accordance with those reported in the literature [29,30]. The increase in the value of zeta potential supports effective loading of ERL within the two dendrimer generations. 

### 2.3. In Vitro Cytotoxicity

**Figure 3:**
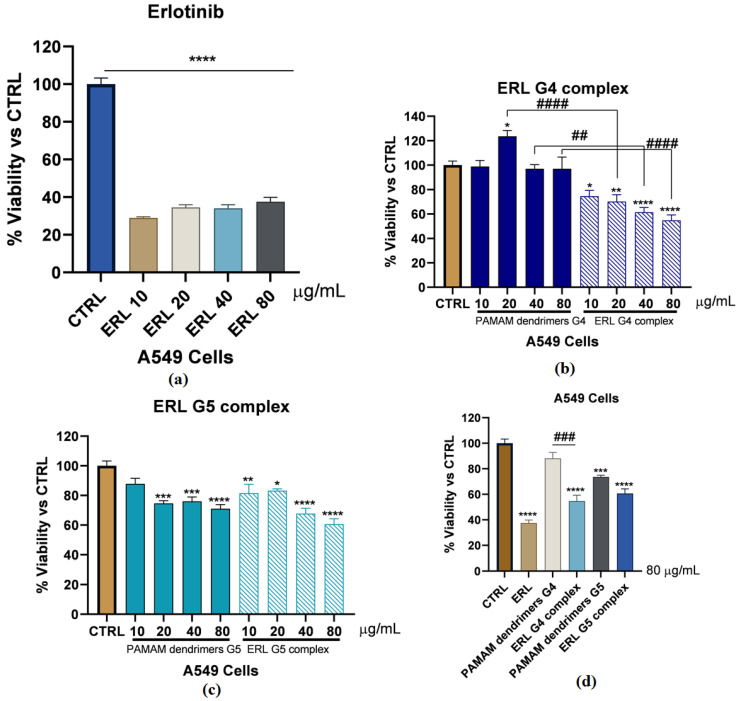
(**a**) MTT results showing Erlotinib decreased the A549 cells’ viability significantly with all the doses (10, 20, 40, 80 µg/mL) (*p* < 0.0001); (**b**) effect of PAMAM dendrimers G4-FITC and ERL-G4-FITC complex after 72 h. Exposure on the cell viability of A549 cells under controlled conditions; (**c**) effect of PAMAM G5-FITC and ERL-G5-FITC complex after 72 h. Exposure on the cell viability of A549 cells under controlled conditions; (**d**) a dose of 80 µg/mL was selected to compare the cell viability between PAMAM dendrimers G4-FITC, ERL-G4-FITC complex, PAMAM dendrimers G5-FITC, and ERL-G5-FITC complex. *—Represents a comparison between each group of treatment to the control (* *p* < 0.05, ** *p* < 0.01, *** *p* < 0.001, **** *p* < 0.0001). #—Represents a comparison between each dose of G4 or G5 PAMAM dendrimer and the matched dose of ERL G4 or G5 complexes (## *p* < 0.01, ### *p* < 0.001, #### *p* < 0.0001).

MTT results have shown that ERL has decreased A549 cells viability significantly with all the doses (10, 20, 40, 80 µg/mL) (*p* < 0.0001) (Figure 3a). Interestingly, PAMAM G4-FITC dendrimers at all the mentioned doses have no significant effect on A549 cells’ viability, while ERL G4-FITC complex (10, 20, 40, 80 µg/mL) exhibited a significant dose-dependent decrease in the A549 cells’ viability in comparison to the control group and the matched doses in blank PAMAM dendrimer G4-FITC groups (20 µg/mL; 49.06%; *p* < 0.0001, 40 µg/mL; 35.61%; *p* < 0.01, and 80 µg/mL; 42.26%; *p* < 0.0001) (Figure 3b). On the other hand, blank PAMAM dendrimer G5-FITC experiments have shown that the blank PAMAM dendrimer G5-FITC at doses of 20, 40, and 80 µg/mL have diminished the viability of A549 cells significantly after 72 h incubation (25.34%; *p* < 0.001, 23.98%; *p* < 0.001, and 28.92%; *p* < 0.0001, respectively). Additionally, ERL-G5-FITC complex at all the mentioned doses have significantly decreased the viability of A549 cells in a dose-dependent manner compared to the control group (18.44%; *p* < 0.01, 16.79%; *p* < 0.05, 32.27%; *p* < 0.0001, and 39.37%; *p* < 0.000, respectively). However, surprisingly there was no significant difference between the PAMAM dendrimers G5-FITC groups and the matched doses in the ERL G5-FITC complex groups (Figure 3c). To facilitate the comparisons between the ERL, blank PAMAM dendrimers G4-FITC, ERL-G4-FITC complex, blank PAMAM dendrimers G5-FITC, and ER-G5-FITC complex, the highest dose of (80 µg/mL) was chosen for all the mentioned groups and the results are illustrated in Figure 3d, concluding that this dose will be considered for further experiments. 

### 2.4. Cellular Uptake Analysis

**Figure 4:**
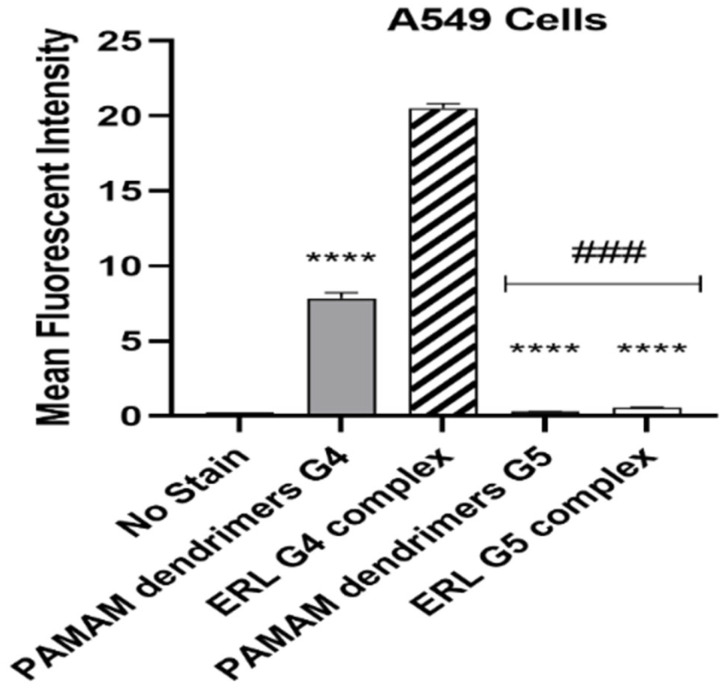
Flow cytometry analysis after treatment for 72 h (*n* = 3/group); **** *p* < 0.0001, ### *p* < 0.001.

The surface bound and internalized particles was measured using flow cytometry analysis (*n* = 3/group) (Figure 4). Following treatment for 72 h, it was concluded that there was a significant uptake of ERL-G4-FITC complex by the cells in comparison to other groups. In addition, we found that the cells showed significant uptake of blank G4-FITC PAMAM dendrimer compared to blank G5-FITC PAMAM dendrimer and PAMAM dendrimer ERL-G5-FITC groups, whereas the G5-FITC plain and PAMAM dendrimer ERL-G5-FITC had minimal uptake by the cells. 

### 2.5. Stability

**Figure 5:**
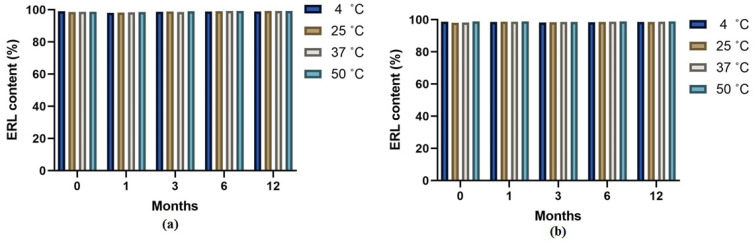
Stability of erlotinib from formula obtained from four different temperatures (4 °C, 25 °C, 37 °C, 50 °C) for (**a**) PAMAM dendrimer G4-FITC complex; (**b**) PAMAM dendrimer G5-FITC complex for a period of 6 months.

ERL was found stable in the samples which were stored in the auto sampler at 4, 25, 37, and 50 °C for 6 months. The ERL contents were measured at 1, 3, and 6 months and results are included in Figure 5. The formulations at different temperatures were found to be stable. The freeze–thaw temperature cycles which were measured for three cycles were also stable.

## 3. Discussion

NSCLC is a type of cancer which does not often respond to therapy. This may be associated with different kinds of mutations in genes such as anaplastic lymphoma kinase (ALK), proto-oncogene B-Raf, discoidin domain receptor tyrosine k 2 (DDR2), and EGFR [31,32]. Upon these mutations, EGFR mutations have the highest rate of incidence, which takes about 10–35% in the exons 18–21 [32]. The NSCLC patients with mutations in the EGFR gene are sensitive to the TKIs such as ERL. Inhibition of the tyrosine kinase domain of EGFR by ERL has been the mainstream treatment for advanced and/or metastatic NSCLC [33]. In fact, many limitations are associated with ERL, especially as the marketed ERL is administered orally as a film-coated tablet. These limitations include side effects, pH dependent solubility, low permeability, drug interaction, and drug resistance. 

These side effects, however, can be reduced by developing a sustained and targeted delivery of ERL. Targeting ERL directly to cancerous cells through the lung may enhance its therapeutic effect [33].

The results showed successful formulations of ERL-loaded PAMAM dendrimer of G4 and G5. Drugs such as ERL could be found encapsulated or conjugated to dendrimers. The surface morphology and size of both generations of G4-FITC and G5-FITC ERL complexes were observed by TEM. It was found that both the plain and loaded dendrimers were found with a distinct spherical morphology. Investigations revealed that the drug–polymer binding showed that ERL was encapsulated inside PAMAM dendrimers and not binding to the PAMAM surface [20]. The mean particle size values of G4-FITC and G5-FITC loaded with ERL were 301.5 ± 8.42 nm and 302.0 ± 8.47 nm, respectively, indicating that the loaded dendrimers are suitable as an inhaler formulation [33,34]. Particle size of the polymer increased after loading of ERL. This may be attributed to the entrapment of ERL molecules within the cavities of PAMAM dendrimers. Sizes of complexes of each generation have enlarged in a range between 100 and 180 nm [20]. In this study, the PDI of empty and loaded PAMAM dendrimers was determined to estimate the average uniformity of the nanocomposite solutions [35]. The values of PDI for blank dendrimers G4-FITC and G5-FITC were 0.05 and 0.300, respectively. After ERL conjugation, the value of PDI changed to 0.02 for ERL-PAMAM dendrimers G4-FITC and G5-FITC. A study by Peng et al. demonstrated that PDI of PAMAM formulation was found to be in a range of 0.23–0.339 [36]. A study conducted by Bielski et al. established triphenylphosphonium (TPP) inside G4-FITC PAMAM dendrimer as an inhaled formulation. They reported a PDI of 0.36, which also supports our results [37]. In a study conducted by Conti et al., where siRNA was introduced into cationic PAMAM dendrimer G4-FITC and was delivered by hydrofluoroalkane (HFA)-based pMDI, the PDI values of these stable dispersions of the dendriplexes were between 0.4 and 0.6, showing satisfactory aerosol physical characteristics and suitability for deep lung deposition, with respirable fractions up to 77% [38]. PDI is related to the size uniformity in nanoformulations. Low PDI values indicated the uniformity in size distribution. However, higher PDI values indicated a large variation in the uniformity of particle size. These results illustrate that no significant changes in PDI were obtained of the dendrimer after loading the dendrimers with ERL. ζ-potential is defined as measuring the charge located on the surface of the particles. An imaginary shear plane outlines the boundary between the diffuse layer ions, which are unaffected by movement of the fluid, and those that are trimmed off by fluid motion. The net charge present at the shear plane of the diffuse layer is quantifiable as zeta potential; higher values of zeta potential indicate a higher rate of repulsion between particles [39]. This characteristic is essential in determining the stability of nanocomposites as well as knowing the intensity of electrostatic attraction between biomolecules and the nanocomposites [40]. The elevation of zeta potential value determines the effective entrapment of ERL within the dendrimers [21,41]. The findings of physicochemical investigations were in accordance with those reported for the PAMAM-dendrimers of neratinib and ruboxistaurin [29,42]. 

The in vitro release dissolution study revealed a prolonged release pattern of ERL from the formulations for more than seven days. According to other studies, it was shown that the slow liberation of drug molecules from dendrimer nanocomposites was due to two main reasons, namely, low aqueous solubility of drug and their confinement within the nanocomposites [42]. The percentage of ERL release from the dendrimers G4-FITC and G5-FITC at pH 5.4 were approximately 70% and 60%, respectively. The medium used pH 5.4, which is a mild acidic pH which mimics the environment around the cancerous cells, and the percentages of ERL release from the dendrimers G4-FITC and G5-FITC at pH 7.4 were approximately 34.97% and 41.9%, respectively. This medium represents extracellular physiological pH [24]. Furthermore, the stability of G4-FITC and G5-FITC ERL nanocomposites were inspected by the eye in order to assess any change in color, precipitation, or turbidity. This was conducted to determine the disintegration of the proposed nanocomposites when exposed to light and/or high temperatures. Results obtained from this study after a specified time show that there was no color change or precipitation detected. Our findings revealed that the nanocomposite was found to be physically stable at 4 °C. The results indicated high formulation stability. The findings of stability studies were in accordance with those reported for the PAMAM dendrimers of ruboxistaurin [29]. A significant increase in cellular uptake of the cationic ERL-G4-FITC PAMAM dendrimer was achieved after 72 h of treatment in comparison to the neutral G5-FITC-ERL dendrimer. Furthermore, in vitro cytotoxicity studies have shown that not only have loaded dendrimers demonstrated a significant decrease in cell viability; dendrimers of both generations G4-FITC and G5-FITC have also. This finding is supported by the hypothesis that dendrimers possess an anticancer effect [43]. The cell viability has decreased significantly by blank dendrimer G4-FITC more than blank PAMAM dendrimer G5-FITC. This is due to the presence of the negative charge on the modified surface of dendrimer G4-FITC in comparison to the neutral charge found on the surface of dendrimer G5-FITC. The main limitation of this work is the efficacy of prepared dendrimer nanocomposites in animal and human models. In the near future, pharmacodynamics and pharmacokinetics studies can be performed in animal and human models to explore the complete potential of developed dendrimer nanocomposites.

## 4. Materials and Methods

### 4.1. Materials

ERL hydrochloride was obtained from Jazeera Pharmaceutical Industries Ltd. (Riyadh, Saudi Arabia). Dialysis cellulose membrane (MWCO 14,000 Da), reusable plastic sample cuvette, and folded capillary ζ-cells were obtained from Sigma Aldrich (St. Louis, MO, USA). Polyamidoamine dendrimer generation 4 (PAMAM dendrimers G4, 1 g vial) and polyamidoamine dendrimer generation 5 (PAMAM dendrimers G5, 1 g vial) were purchased from Nanosynthons (Mt. Pleasant, MI, USA). The HPLC-PDA purity of PAMAM dendrimers G4 and G5 is included in Figure S5. All chemicals and reagents were of analytical grade.

### 4.2. Instrumentation and Chromatographic Conditions

The analysis of ERL was performed at 25 ± 1 °C by means of Waters HPLC system 1515 isocratic (Waters, Milford, MA, USA) pump, 717 auto sampler, a programmable UV–Visible variable wavelength detector, a column oven, a SCL 10AVP system controller, and an inline vacuum degasser was used. The HPLC system utilized the use of the Millennium software, version 32, for data processing and analysis. The column utilized for this analysis was a Nucleodur (150 mm × 4.6 mm) RP C18 with particle size of 5 μm. The eluent/solvent system was composed of methanol: water (5:1 % *v/v*). The solvent system flowed with a flow rate of 1.0 mL/min. The detection of ERL was carried out at 254 nm. The samples (20 μL) were introduced into the system via a Waters auto sampler. The suggested method was validated for linearity, accuracy, precision, robustness, and sensitivity.

### 4.3. Synthesis of Dendrimers of ERL in G4-FITC PAMAM Dendrimer and G5-FITC PAMAM Dendrimer

The lyophilized form of PAMAM dendrimers (G4 and G5) were dissolved in sterilized Milli-Q deionized water (10% PAMAM). Each generation was mixed separately with excess amount of ERL solution in a molar ratio of 25:1 of ERL to dendrimer. The 25:1 molar ratio of ERL:denfrimer was selected based on our previous studies with ruboxistaurin [29]. In our previous studies, various molar ratios of drug:dendrimer were investigated and 25:1 ratio was found to be the best. As a result, this ratio was selected in this work [29]. The mixtures were stirred overnight at room temperature for equilibration. Thereafter, the amount of ERL attached on the dendrimer was removed by placing it in cellulose membrane (MWCO14,000 Da) and dialyzing it against deionized water for 24 h to eliminate unconjugated or free ERL. The mixtures were lyophilized for 60 h [42]. Briefly, G4 and G5 PAMAM dendrimers were dissolved in deionized water and then mixed with FITC (E-Merk, Darmstadt, Germany) in 1:6 molar ratios. The components of dendrimers are expected to dissolve FITC in dendrimers. Then the formulations were stirred for 24 h [42].

### 4.4. Morphological Studies, TEM

TEM imaging was performed to illustrate the morphological characteristics of the loaded dendrimer (size and shape of the particles) and compared with the plain dendrimer. The blank and loaded dendrimers were visualized by TEM (JEM1230EX; Tokyo, Japan). A drop of each blank and loaded dendrimer was placed onto a grid and set aside for air drying for 15 min then images of the particles was taken by TEM [42].

### 4.5. Characterization of ERL Loaded Dendrimer

The surface potential of different nanocomposites was evaluated by Malvern Zetasizer Nano-ZS (Malvern Instruments Ltd., Malvern, UK). Samples were suspended in water or PBS (pH 5.4 and pH 7.4). They were under analysis at 25 °C. The hydrodynamic diameter of the nano conjugates was also assessed using a Malvern Zetasizer Nano-ZS (Malvern Instruments Ltd.).

### 4.6. Particle Size Distribution, PDI, and ζ-Potential

The two generations of nano conjugates were tested to characterize the physicochemical properties, which include particle size distribution, zeta potential, and PDI. The size of particles was assessed according to light scattering and the data were analyzed by an attached software, giving a measure of distribution of particle size. The particle size was measured at neutral pH (water). For the measurement, one mL of each formulation was diluted 100 times with water and subjected to particle size measurement. The ζ-potential was measured at PBS pH 5.4 and 7.4. For ζ-potential measurement, one mL of each formulation was diluted 100 times with each buffer and subjected for the ζ-potential measurement. ζ-potential determined the net charge presented on particles, which were moving in an electric field [42]. 

### 4.7. Drug Loading, Entrapment Efficiency

Approximately 0.002 g of G4-FITC-ERL and G5-FITC-ERL PAMAM dendrimer were dissolved in Milli-Q deionized water and placed in cellulose membrane (MWCO 14,000). Then, they were placed in 100 mL medium of deionized water. The mixture was vortexed for 1 h at 100 RPM. After overtaxing, 5 mL of solution was analyzed by HPLC to determine the amount of free drug which was not entrapped in the nano formulation. The following equations were used to measure the PAMAM dendrimer EE and ERL DLC:

(1) $$\mathrm{EE}\;(\%)=\frac{Total\;drug-Free\;drug}{Total\;drug}\times 100;$$

(2) $$\mathrm{DLC}\;(\%)=\frac{Drug\;weight\;in\;nanoparticles\;}{Nanoparticles\;weight}\times 100.$$

### 4.8. In Vitro Drug Release Studies Using a Dialysis Method in PBS (pH 5.4 and pH 7.4)

In vitro drug release studies were performed using the dialysis method [42]. An aliquot (5 mL) of each individual sample was placed in cellulose membrane dialysis tubing (MWCO 14,000). The mixtures were subjected to rotary machine stirring at 100 RPM in 50 mL of 40% methanol PBS pH (5.4) and pH (7.4) media at 37 ± 0.5 °C. At various time intervals, an aliquot of (3 mL) was withdrawn from the released media and was replaced with an equivalent volume of fresh media. Triplicate samples (*n* = 3) of each PAMAM generation dendrimer were measured. Meanwhile, the in vitro dissolution study for the reference tablet was examined using Pharma test (DT 70, Germany) dissolution apparatus, utilizing type II apparatus (paddle) at stirring speed of 75 RPM in 1000 mL of 0.01 N HCl, and compared with 0.02% Tween 80 in 0.01 N HCl [44]. The amount of ERL released from each medium was then analyzed with HPLC-UV [24].

### 4.9. Cell Culturing and Cytotoxicity Assay

Non-small lung carcinoma (A549) cells were cultured at a density of ~ 3 × 10^5^ cells/mL in basal medium containing 1:1 Dulbecco’s modified Eagle’s medium 1× (DMEM 1×): 10% fetal bovine serum (FBS); and 1% streptomycin/penicillin (100 μg/mL and 100 Units/mL, respectively). Cells were maintained in a humidified incubator containing 5% CO_2_ at 37 °C. Once cells became confluent, they were passaged by trypsinization (300 μL) into T-75 flasks. Cells from passages 3 to 10 were utilized to conduct all proposed experiments. Ninety six well plates were utilized to perform MTT assay. All treatments were accomplished in full culture media containing FBS and streptomycin/penicillin. After every 48 h, the medium was replaced, regardless of the presence or absence of various treatments. MTT assay is considered a colorimetric process conducted to assess both cell proliferation and survival. This assay was used to evaluate the cytotoxicity of either ERL, ERL G4-FITC complex, ERL G5-FITC complex, or vehicles alone (PAMAM dendrimers G4-FITC or PAMAM dendrimers G5-FITC) on A549 cells. Cells were cultured at the conditions described above. Then, cells were harvested and trypsinized to be counted. Additionally, 1 × 10^4^ cells per well in 96 wells plates were seeded. The plates were incubated for 24 h. In the survival experiments, viability was assessed in cells incubated with either ERL, ERL-G4-FITC complex, ERL-G5-FITC complex, or vehicles alone (10, 20, 40, and 80 µg/mL) for 72 h. Once cells were incubated at the proposed conditions, 10 μL of MTT (5 mg/mL PBS) reagent was added to each individual well for 30 min until purple precipitate was viable. Then, we added 100 μL of DMSO at room temperature while shaking for 5 min. At the end, absorbance at 570 nm was documented by using a micro-plate reader.

### 4.10. Cellular Uptake Analysis

A549 cells were seeded in 12-well plates at 1 × 10^5^ cells per well. After cells had attached and proliferated for 24 h, the culture medium was replaced with fresh media and treated with FITC-labeled dendrimers at a concentration of 80 µg/mL for 72 h. Treated cells were washed three times with PBS then harvested by trypsinization. Following that, the cells were collected, centrifuged, and suspended in 500 µL of PBS. Then, the cells were analyzed by flow cytometry (Cytomics FC 500; Beckman Coulter, CA, USA). Mean fluorescence intensities (MFI) of different cell exposure groups were compared and analyzed. 

### 4.11. Stability Studies

Accelerated stability studies were performed in this work. Hence, the stability studies were conducted for 1, 3, and 6 months at accelerated temperatures of 4, 25, 37, and 50 °C. The stability of the tested samples was expressed as a percentage recovery relative to the freshly prepared solution. All stability studies were conducted in triplicate. Lastly, freeze/thaw stability tests were assessed on both formulations, after they had been frozen at (−30 °C) then thawed at room temperature for three cycles. The drug content was measured at different temperatures of storage [42].

### 4.12. Statistical Analysis

Diverse physicochemical variables, drug delivery and biological data were analyzed using Graph Pad InStat^®^ software (San Diego, CA, USA) by the student *t*-test for two groups and aone-way analysis of variance (ANOVA) for multiple groups. Differences between each of the two related parameters were statistically significant for a *p*-value of equal or less than 0.05.

## 5. Conclusions

Erlotinib is an antineoplastic agent that has been marketed since 2004 as a film-coated tablet for the treatment of non-small cell lung cancer and pancreatic cancer. There are a vast number of obstacles to the current marketed erlotinib being delivered effectively to the cancerous cells; these include pH dependent solubility, inter-patient variability, and drug-drug interaction. To overcome these limitations, a non-invasive therapy of erlotinib was designed. This was performed utilizing nanocomposites of PAMAM dendrimers. Additional assessments of the formulations, including in vitro characterization, were conducted to ensure the feasibility of the formulation to be administered as a targeted inhaled drug delivery system. The physicochemical investigations suggested the proper formation of PAMAM dendrimers. The drug release studies showed prolonged release of erlotinib from both generation dendrimers. However, G4-FITC dendrimers showed higher drug release compared to the G5-FITC dendrimers. The dendrimer nanocomposites were found to be stable at 4 °C. Cytotoxicity studies revealed the suitability of dendrimer nanocomposites in the treatment of NSCLC. The proposed nanocomposites will overcome the unwanted adverse events, low bioavailability, and resistance associated with the currently marketed orally administered erlotinib tablet, and they will increase the efficacy by ensuring the sustained exposure of the cancerous cells to erlotinib. As a result, the frequency of administration of erlotinib will be remarkably reduced.

## Figures and Tables

**Table 1:** Physicochemical characteristics of blank and ERL-loaded PAMAM dendrimer G4-FITC and G5-FITC. The particle size and polydispersity index (PDI) were measured. Data represent averages ± SD, (*n* = 3).

ERL-PAMAM Nanocomposites	Particle Size (nm) ± SD	PDI ± SD	EE (%)	DLC (%)
Blank dendrimer G4-FITC	200.0 ± 3.12	0.05 ± 0.00	-	-
ERL-loaded dendrimer G4-FITC	301.5 ± 8.42	0.02 ± 0.00	99.96	14.05
Blank dendrimer G5-FITC	224.8 ± 22.4	0.30 ± 0.01	-	-
ERL-loaded dendrimer G5-FITC	302.0 ± 8.47	0.02 ± 0.00	99.92	7.62

## Data Availability

This study did not report any data.

## Supplementary Materials

The following supporting information can be downloaded at: https://www.mdpi.com/article/10.3390/molecules28093974/s1. Figure S1: TEM images at low magnification to inspect the morphology of (a) blank G4-FITC PAMAM dendrimers; (b) erlotinib conjugated G4-FITC PAMAM dendrimers; (c) blank G5-FITC PAMAM dendrimers; and (d) erlotinib conjugated G5-FITC PAMAM dendrimers; Figure S2: Particle size distribution by intensity for (a) blank G4-FITC PAMAM dendrimers; (b) erlotinib conjugated G4-FITC PAMAM dendrimers; (c) blank G5-FITC PAMAM dendrimers; and (d) erlotinib conjugated G5-FITC PAMAM dendrimers; Figure S3: Zeta potential measurements for (a) blank G4-FITC PAMAM dendrimers; (b) erlotinib conjugated G4-FITC PAMAM dendrimers; (c) blank G5-FITC PAMAM dendrimers; and (d) erlotinib conjugated G5-FITC PAMAM dendrimers at pH 5.4; Figure S4: Zeta potential measurements for (a) blank G4-FITC PAMAM dendrimers; (b) erlotinib conjugated G4-FITC PAMAM dendrimers; (c) blank G5-FITC PAMAM dendrimers; and (d) erlotinib conjugated G5-FITC PAMAM dendrimers at pH 7.4; Figure S5: HPLC-PDA purity of polyamidoamine (PAMAM) dendrimers G4 and PAMAM dendrimers G5.

## Sample Availability

Samples of the compounds erlotinib are available from the authors.
