# Supplementary material for: Erlotinib-Loaded Dendrimer Nanocomposites as a Targeted Lung Cancer Chemotherapy
Source: Molecules. 2023 May 8;28(9):3974. doi: 10.3390/molecules28093974 (PMC10180382; doi:10.3390/molecules28093974)
Supplement: Supplementary file 1 [file molecules-28-03974-s001.zip › molecules-2263743-supplementary.pdf]

Supplementary Materials

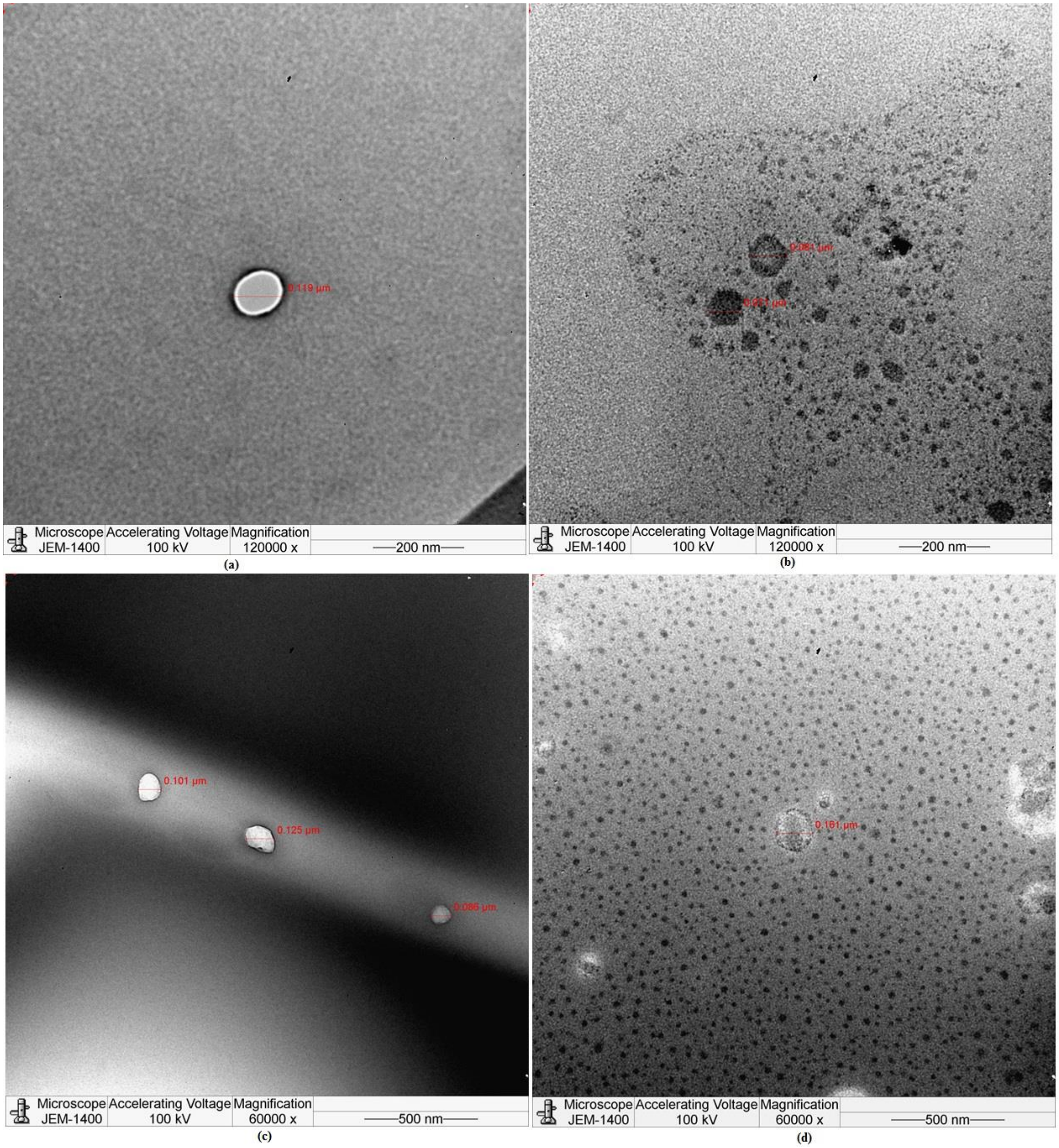

Figure S1. TEM images at low magnification to inspect the morphology of (**a**) blank G4-FITC PAMAM dendrimers; (**b**) erlotinib conjugated G4-FITC PAMAM dendrimers; (**c**) blank G5-FITC PAMAM dendrimers; and (**d**) erlotinib conjugated G5-FITC PAMAM dendrimers.

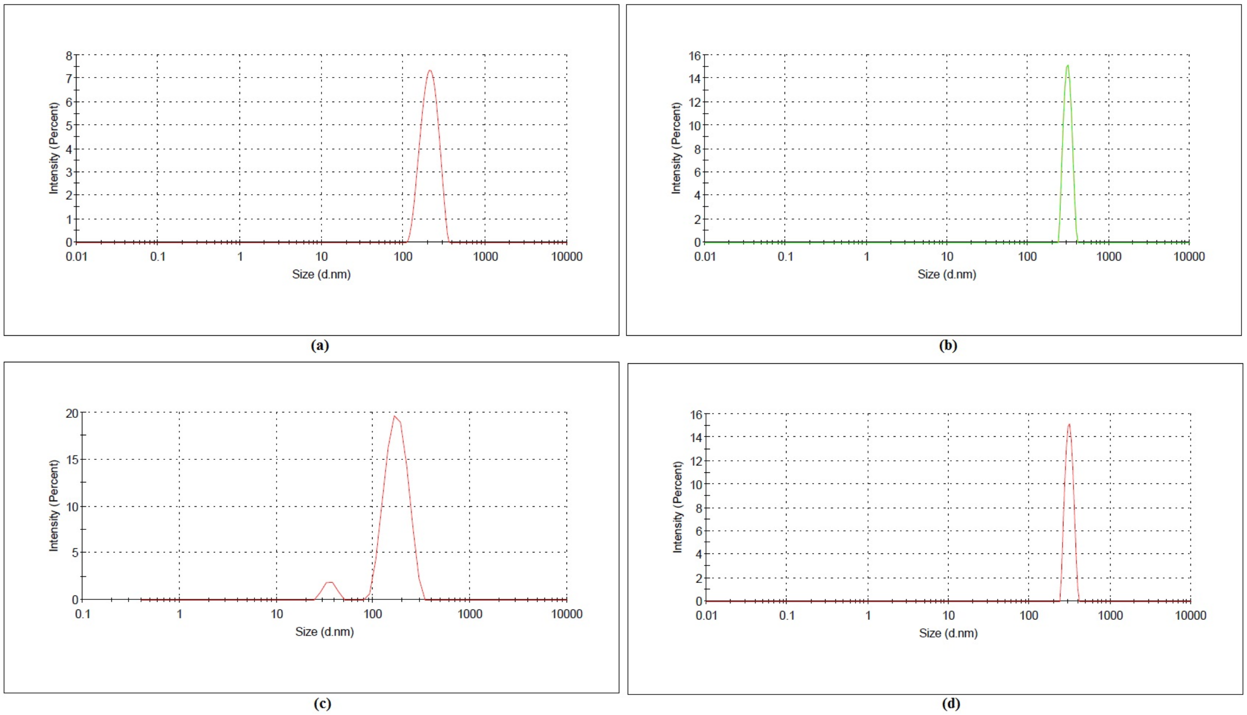

Figure S2. Particle size distribution by intensity for (**a**) blank G4-FITC PAMAM dendrimers; (**b**) erlotinib conjugated G4-FITC PAMAM dendrimers; (**c**) blank G5-FITC PAMAM dendrimers; and (**d**) erlotinib conjugated G5-FITC PAMAM dendrimers.

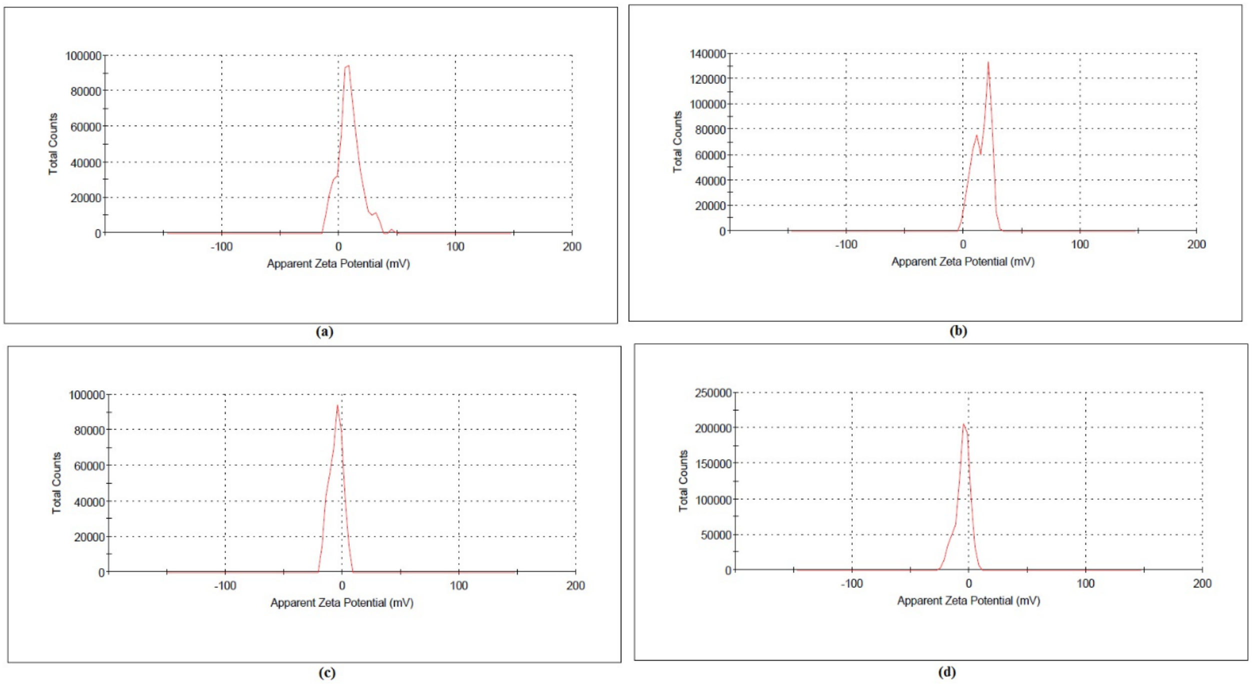

Figure S3. Zeta potential measurements for (**a**) blank G4-FITC PAMAM dendrimers; (**b**) erlotinib conjugated G4-FITC PAMAM dendrimers; (**c**) blank G5-FITC PAMAM dendrimers; and (**d**) erlotinib conjugated G5-FITC PAMAM dendrimers at pH 5.4.

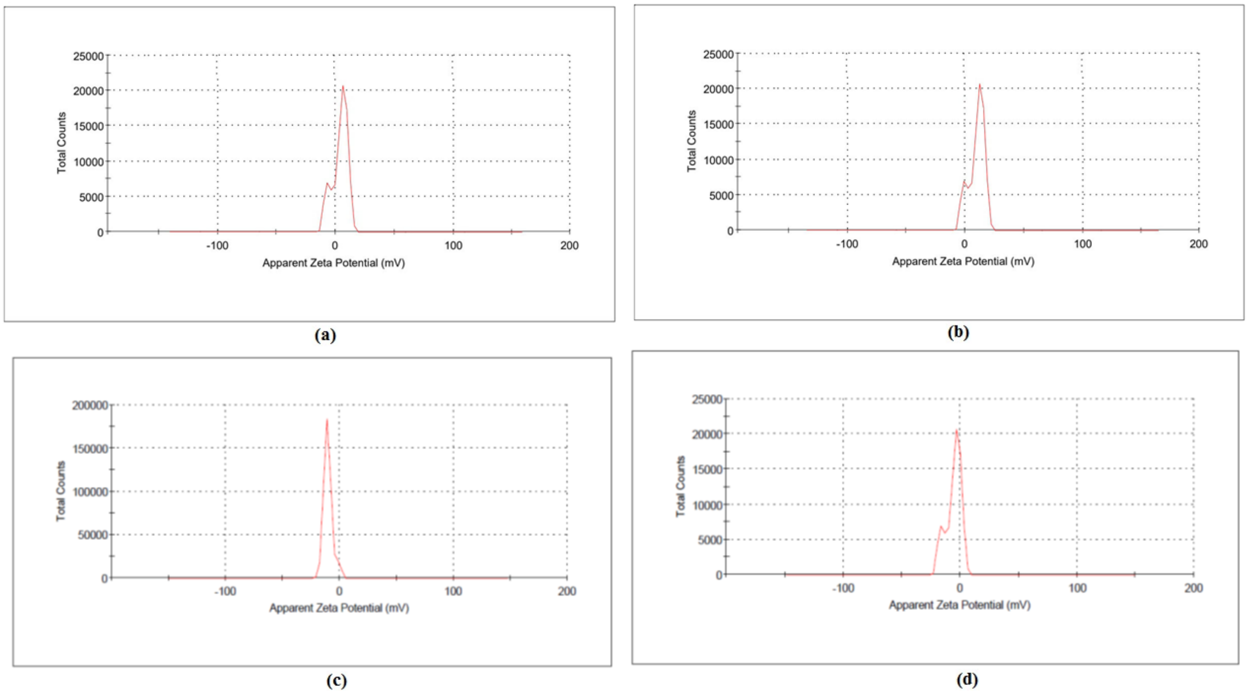

Figure S4. Zeta potential measurements for (**a**) blank G4-FITC PAMAM dendrimers; (**b**) erlotinib conjugated G4-FITC PAMAM dendrimers; (**c**) blank G5-FITC PAMAM dendrimers; and (**d**) erlotinib conjugated G5-FITC PAMAM dendrimers at pH 7.4.

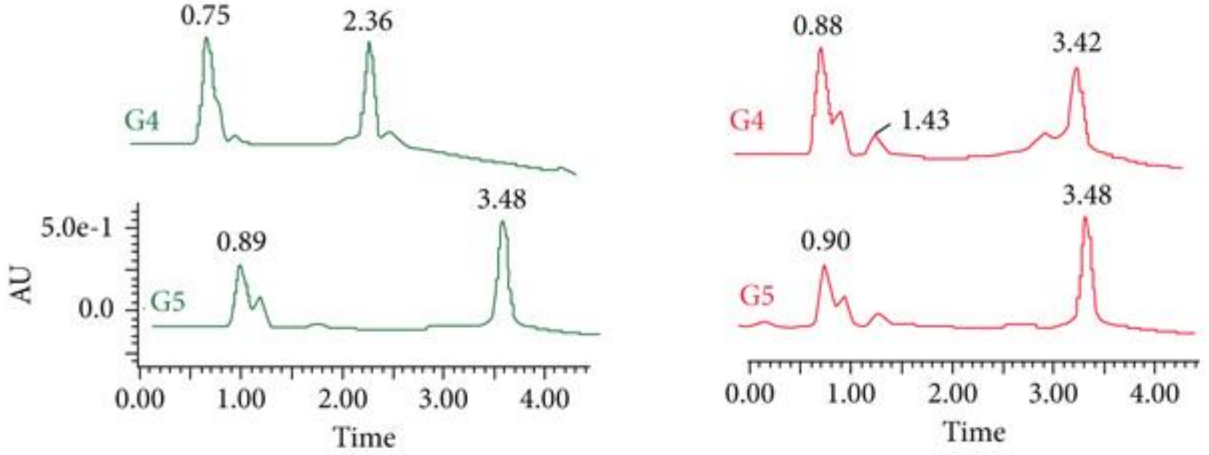

Figure S5. HPLC-PDA purity of polyamidoamine (PAMAM) dendrimers G4 and PAMAM dendrimers G5.
